# Sequencing-Based Approaches Reveal Low Ambient Temperature-Responsive and Tissue-Specific MicroRNAs in *Phalaenopsis* Orchid

**DOI:** 10.1371/journal.pone.0018937

**Published:** 2011-05-06

**Authors:** Feng-Ming An, Shuan-Rung Hsiao, Ming-Tsair Chan

**Affiliations:** 1 Institute of Biotechnology, College of Bioscience and Biotechnology, National Cheng Kung University, Tainan, Taiwan; 2 Biotechnology Center in Southern Taiwan, Academia Sinica, Tainan, Taiwan; 3 Institute of Tropical Plant Sciences, College of Bioscience and Biotechnology, National Cheng Kung University, Tainan, Taiwan; 4 Agricultural Biotechnology Research Center, Academia Sinica, Taipei, Taiwan; University of Melbourne, Australia

## Abstract

Plant small RNAs (smRNAs) are short, non-coding RNA molecules that mediate RNA silencing and regulate a group of genes involved in plant development and responses to environmental stimuli. Low temperature is necessary to initiate stalk development in the orchid *Phalaenopsis aphrodite* subsp. *formosana*. To identify smRNAs in *Phalaenopsis* responding to low temperatures, a smRNA profiling analysis using high-throughput sequencing technology was performed. Subsequent bioinformatic analysis was applied to categorize the miRNAs identified. A total of 37,533,509 smRNA reads yielded 11,129 independent orchid miRNA sequences, representing 329 known miRNA families identified in other plant species. Because the genomic resources available for *Phalaenopsis* are limited, a transcriptomic database was established using deep sequencing data sets to identify miRNAs precursors and their target transcripts. Comparing small RNAs and the transcriptomic database, 14 putative miRNA precursors of 10 miRNA families were identified, as were hundreds of putative targets. Comparing sequencing data and smRNA northern hybridization results identified miR156, miR162, miR528 and miR535 as low temperature-induced miRNAs. In addition, tissue-specific expression of these miRNAs was investigated. It was concluded that miR156 and miR172 may be components of a regulatory pathway mediating transition from the vegetative to the reproductive phase in *Phalaenopsis*. The smRNA and transcriptomic databases could be the foundations for further research aimed at elucidating the control of the flowering time in orchids.

## Introduction

Small non-protein-coding RNAs (smRNAs) present in plants are 20–24 nucleotides (nt) in length and carry out diverse functions during plant development in response to environmental stimuli, in terms of anti-viral defense and involvement in epigenetic modifications [1]. Endogenous smRNAs regulate transcriptional and post-transcriptional gene silencing through DNA methylation, chromatin modification, mRNA cleavage/degradation and translational inhibition [2]. On the basis of precursor structures and biosynthetic pathways, plant smRNAs are broadly classified as small interfering RNA (siRNA) or microRNA (miRNA). The microRNA (MIR) pathway produces microRNA (miRNA), the trans-acting siRNA (TAS) pathway produces trans-acting small interfering RNA (ta-siRNA), the post-transcriptional gene silencing (PTGS) pathway produces virus-induced (VI-PTGS) and repeat associated siRNA (rasiRNA), and the natural antisense transcripts (NATs) pathway produces naturally occurring siRNAs (nat-siRNA) [3].

Transcripts transcribed from *MIR* genes, usually located between coding genes, are termed “pri-miRNAs”. Pri-miRNAs are processed by DICER-LIKE1 (DCL1) proteins to generate stem–loop precursor miRNAs, termed “pre-miRNAs”. Final processing of these precursors is carried out by a macromolecular protein complex formed by DCL1, HYPONASTIC LEAVES1 (HYL1) and SERRATE (SE), and they are exported to the cytoplasm through the action of the plant exportin 5 ortholog HASTY. Ultimately, the guide miRNA strand is incorporated into ARGONAUTE (AGO) proteins to initiate the downstream gene silencing process [4].

*Phalaenopsis* orchids are appreciated for their beauty and represent a commercialized species with high economic value. *Phalaenopsis aphrodite* subsp. *formosana*, a native species of *Phalaenopsis* in Taiwan, has been widely used for breeding hybrids with various colors, shapes and sizes of floral organs. Industries often use high temperatures (>28°C) to inhibit spike initiation and low temperatures (24°C/18°C, day/night) to synchronize the flowering date in certain *Phalaenopsis* species. Without prolonged exposure to low ambient temperatures, spike initiation would be inhibited. Several studies have demonstrated that low temperatures during the day or at night are necessary for *Phalaenopsis* orchids to flower [5], [6]. *Phalaenopsis* orchids are classified biologically as crassulacean acid metabolism (CAM) plants. Metabolic analysis based on the characteristics of CAM plants elucidated the relationship between the metabolic pool and low temperature conditions for spike induction in *Phalaenopsis aphrodite* subsp. *formosana* [6]. Some studies have demonstrated the importance of low ambient temperature requirements for the reproductive phase transition in *Phalaenopsis aphrodite* subsp. *formosana*, but the regulatory mechanism(s) underlying this transition have yet to be elucidated.

Several studies have demonstrated that miRNAs are involved in abiotic and biotic stresses such as oxidative stress [7], cold [8], [9], salt, nutrient deficiency [10], [11], [12] and pathogen infection. miRNA398 is a repressor of Cu-Zn superoxide dismutase genes (At1g08830, *CSD1*; At2g28190, *CSD2*), which act as ROS scavengers [7]. In response to oxidative stress miR398 is down-regulated, resulting in an immediate increase in the level of *CSDs*, which remove ROS. miR395 and miR399 regulate gene expression to maintain sulfur and phosphate homeostasis during nutrient-deficient conditions. ATP sulfurylases (At3g22890, *ASP1*; At4g14680, *ASP3*; and At5g43780, *ASP4*) and sulfate transporters (At5g10180, AtSULTR2;1), which are involved in sulfur assimilation and translocation, specifically, are targeted by miR395 [11], [12]. miR399 is up-regulated during phosphate starvation, causing repression of its target gene, *PHO2* (also named *UBC24*); as a result, Pi uptake is increased [10]. Several studies have analyzed the expression patterns of miRNAs under conditions of various stresses using deep sequencing and/or miRNA microarray. These studies identified the putative miRNA targets, but require validation, and the overall pathways have yet to be determined. Temperature-responsive smRNAs were identified predominantly under temperature stress conditions. In response to cold stress, miR156/157, miR159/319, miR165/166, miR169, miR172, miR393, miR394, miR396, miR397 and miR398 were up-regulated [9]. Low ambient temperature conditions are less stressful than extreme cold. Two miRNA families, miR156 and miR169, were up-regulated at low ambient temperatures; validation was achieved by comparing northern hybridization results with those obtained using microarray analyses [13]. However, the regulatory roles of smRNAs that exhibit a change in their expression profiles during low ambient temperature conditions in other species remains unclear.

To investigate which functional miRNAs potentially affect the development and flowering of *Phalaenopsis aphrodite* subsp. *formosana* during low ambient temperature conditions, candidate miRNA species were identified using deep sequencing. After aligning these candidates with known miRNAs found in other plant species, it was evident that some miRNA families were highly represented including miR156, miR166, miR167, miR168, miR172, miR528 and miR535. By mining the sequencing data, several abundant miRNAs were shown to be induced by low temperature treatment; the expression profiles of these miRNAs were confirmed using northern hybridization. The results demonstrated that the four major miRNA families were induced by low temperatures and that their expression was tissue specific. To identify target transcripts of miRNAs, referenced genomic or EST (expressed sequence tag) sequences should be available. *Phalaenopsis* has few sequence resources. Therefore, the usability of a transcriptomic database established by 454 pyrosequencing was investigated as a possible reference. Several target transcripts were annotated to known miRNA-mediated target genes in other plant species, revealing conservation of miRNA regulatory pathways. Furthermore, some of these target transcripts were highly associated with phase transition and flower development, suggesting the possibility of regulatory pathways for low temperature-responsive miRNAs. Previous studies have demonstrated that specific miRNAs are involved in the regulation of development and flowering in plants. For example, the sequential action of ath-miR156 (ath-, *Arabidopsis thaliana*) and ath-miR172, which leads to repression of *SMZ*, *SNZ*, *TOE1* and *TOE2*, regulates flowering in *Arabidopsis* [14], [15]. Therefore, miR156, miR172 and their target transcripts were selected as candidates to validate the entire approach, suitable for investigating miRNA-mediated regulatory pathways.

In this study, two comprehensive smRNA and transcriptomic databases have been developed, which not only contribute to the understanding of low-temperature regulation but may also prove useful tools for future research projects. The results presented herein provide an insight into the regulatory role of miRNAs in *Phalaenopsis aphrodite* subsp. *formosana* in response to low ambient temperatures and provide valuable information in relation to the control of flowering time.

## Results

### The microRNA biosynthesis pathway in *Phalaenopsis aphrodite* subsp. *formosana* is similar to that of *Arabidopsis*

To identify the existing miRNA biosynthesis pathways in *Phalaenopsis aphrodite* subsp. *formosana*, Blast searches against the deep sequencing-based *Phalaenopsis* transcriptomic database were performed using 29 known *Arabidopsis* genes [3] involved in miRNA biosynthesis. With the exception of *AGO7* and *SUVH4*, miRNA biosynthesis components of *Arabidopsis* were identified in the native orchid. This reveals a high degree of conservation of these pathways between *Arabidopsis* and *Phalaenopsis* (Table S1).

### Small RNA sequencing using Illumina

**Figure 1:**
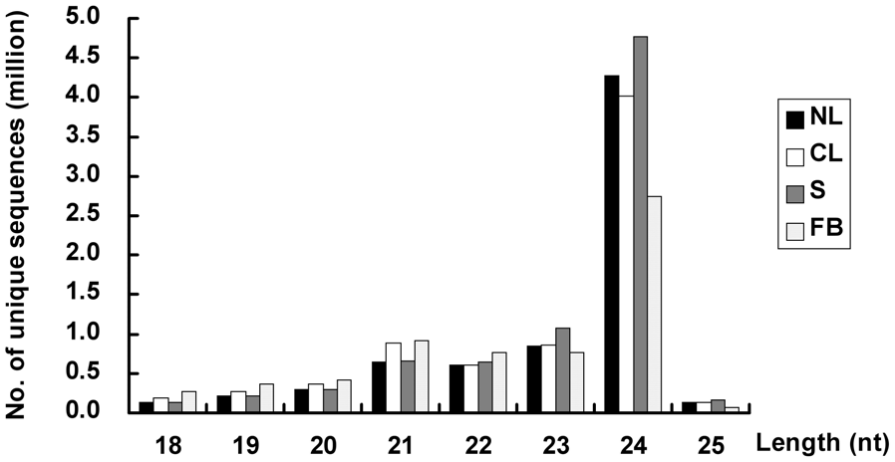
Size distribution of small RNA sequences in the separated tissues of native *Phalaenopsis*. NL: leaves from untreated plants, CL: leaves after low ambient temperature treatment, S: stalks, FB: flower buds.

Leaves of *Phalaenopsis aphrodite* subsp. *Formosana*, with or without low temperature treatment, were harvested for smRNAs expression analysis, and stalks and flower buds were harvested for investigating tissue-specific expression of smRNAs. Four pools of smRNAs were generated and subjected to next-generation sequencing using Illumina technology (see Methods). After excluding polyA sequences, short reads with lengths below 18-nt and contaminants of adapter and null sequences, 28,932,901 from a total of 37,533,509 reads contained correct adapter sequences (Table 1). Data analysis revealed that the four batches contained a similar size distribution of smRNAs, with sequences of 24-nt representing the most abundant class in each batch. Although the number of total reads was similar for each batch, the number of clean reads was reduced in the batch containing flower buds. There were more reads corresponding to 21-nt-long smRNAs in low ambient temperature-treated leaves and flower buds than in reads of untreated leaves and stalks (Figure 1). The number of sequences with a length of 24-nt was lower in the group of flower buds (∼2.8 million) than in all other batches (approximately four million, on average). After removal of rRNA, tRNA, snRNA, snoRNA and repeat sequences characterized in Rfam [16] and Genbank [17], a total of 3.8–5.3 million unique sequences in the range of 18–25 nt remained in the four batches and these were used for further analyses (Table 1).

**Table 1:** Summary of small RNA sequencing.

	NL	CL	S	FB
Number of sequence reads	9,012,431	9,621,208	9,891,244	9,008,626
high quality	7,655,780	8,188,034	8,557,683	7,816,982
adapter3 null	6,011	6,207	11,668	5,478
insert null	1,412	1,483	1,902	58,600
adapter5 contaminants	32,889	39,121	49,417	61,064
smaller than 18 nt	413,178	741,172	478,813	1,373,976
polyA	665	1,020	809	693
clean reads	7,201,625	7,399,031	8,015,074	6,317,171
No. of unique sequences	4,633,037	4,386,295	5,330,904	3,853,304
miRNA	4,207	4,981	3,288	4,831
rRNA	32,280	37,938	29,959	27,394
repeat	742	929	911	1,137
snRNA	456	463	570	628
snoRNA	354	357	416	463
tRNA	6,446	7,600	4,784	4,901
unann	4,577,057	4,323,265	5,275,951	3,803,023

### Alignment to known miRNAs

**Figure 2:**
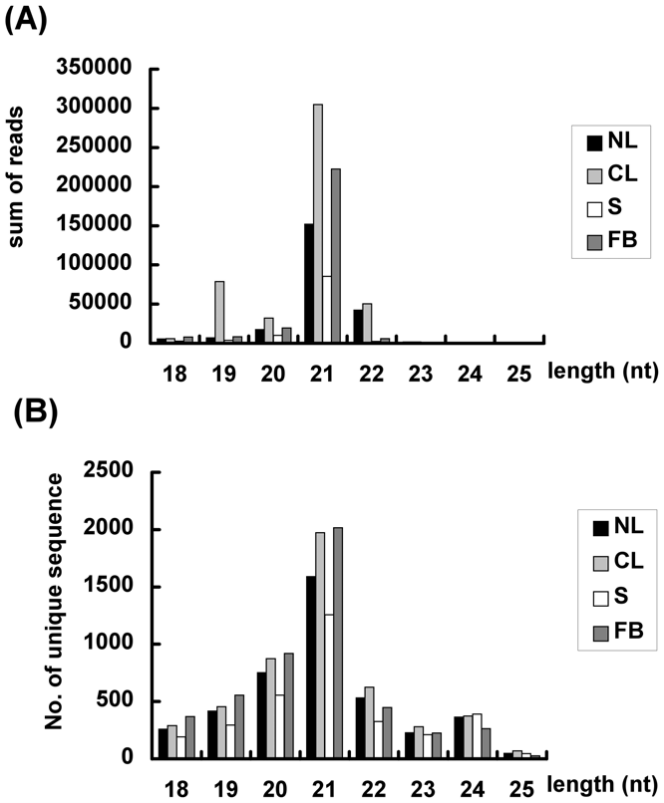
Characterization of conserved miRNAs in various tissues of *Phalaenopsis* orchid. Size distribution of mature miRNAs in each tissue as a function of the sum of reads (A) and the number of unique sequences (B) NL: leaves from untreated plants, CL: leaves from low ambient temperature treatment, S: stalks, FB: flower buds.

**Figure 3:**
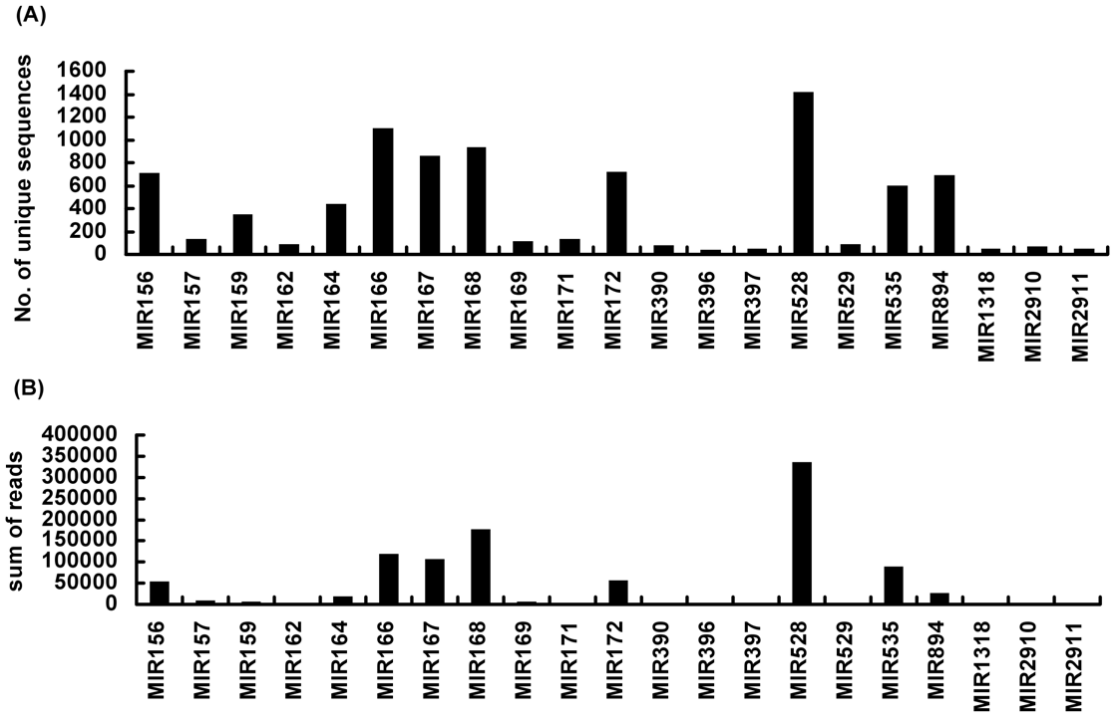
Characterization of conserved miRNA families. miRNA families pertaining to the number of unique sequences (A) and to the sum of reads in an individual miRNA family (B). NL: leaves from untreated plants, CL: leaves from low ambient temperature treatment, S: stalks, FB: flower buds.

The unique sequences of the four batches were used for aligning to known miRNA sequences from miRBase v15; between 3,300 and 5,000 sequences were obtained from various samples (Table 1). The size distribution of match-to-known miRNA sequence reads in *Phalaenopsis* orchid demonstrated a maximum at miRNAs 21-nt in length (Figure 2), consistent with data obtained previously in another plant species using a similar approach [18]. Alignment of miRNA sequences of the four samples revealed the presence of 11,129 unique sequences belonging to 329 miRNA families. Within these 329 miRNA families, the main components in *Phalaenopsis* orchid were miR156, miR159, miR164, miR166, miR167, miR168, miR172, miR528, miR535 and miR894 (Figure 3A). The more abundant miRNA families contained hundreds of unique sequences (Figure 3B). A low number of sequence reads may be insufficient to represent the expression pattern of a single sequence. Therefore, sequences with a high number of reads were used to study the roles of low temperature-responsive miRNAs in the orchid *Phalaenopsis*.

### Sequencing-based analysis of low ambient temperature-responsive miRNAs in *Phalaenopsis aphrodite* subsp. *formosana*

**Figure 4:**
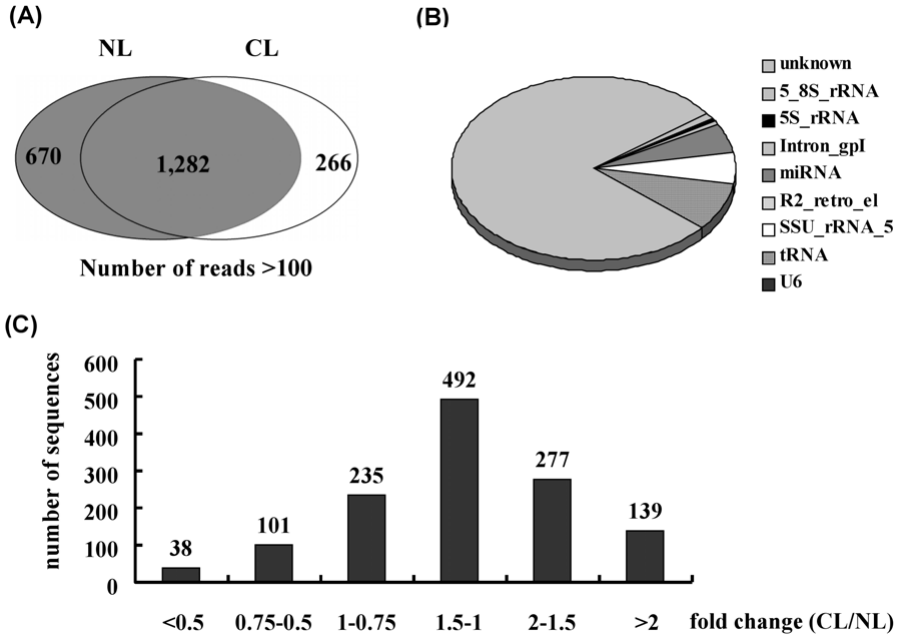
Summary of low temperature-responsive miRNA selection. Abundant smRNAs from untreated and low temperature-treated leaves (A); Characterization of abundant smRNAs (B); Fold change of small RNA unique sequences by comparing the dataset of the low temperature treated group to the untreated group (C). NL: leaves from untreated plants, CL: leaves from low ambient temperature treatment.

For the analysis of low temperature responsive smRNAs in the orchid *Phalaenopsis*, smRNA sequences with a number of reads over 100 were considered highly abundant. By comparing abundant smRNAs in the group of untreated leaves with that of low temperature-treated leaves, 1,282 unique sequences present in both groups were identified (Figure 4A). Among these sequences, 65 were miRNAs (5.1%), 107 were tRNAs (8.3%), 93 were rRNA (7.3%) and 1,008 were unknown sequences (78.6%) (Figure 4B). A calculation of fold change of reads (fold = reads of treated group/untreated group) for these sequences revealed that 416 smRNA sequences in the low temperature-treated group had a >1.5 fold increase in reads, whereas 137 smRNA sequences had a decreased ratio (Figure 4C). Further analysis indicated that miR156, miR159, miR162, miR168, miR396, miR528, miR535 and miR894 were induced in leaves during low ambient temperature treatment; miR172 demonstrated a repressed pattern in leaves during the same treatment. miR164, miR166, miR167 and miR390 were expressed at similar levels under low temperature-treated or untreated conditions (Table 2).

**Table 2:** List of predicted precursors.

MIRNA loci	miRNA seq	length	predicted precursor*	MFE (kcal/mol)
miR156	UGAGAGAGAAAGAGAGAGAGCAU	23	con_contig16056	−302.42
miR164	UGGAGAAGCAGUGCACGUGUU	21	con_contig07032, chi_contig01076	−42.5, −44.9
miR171	AUGAGCCGAACACGAACACACU	22	con_contig03214	−24.9
	UUGAGCCGAACACGAACACACC	22		
miR396	UCCCACAGCUUUCUUGAACUG	21	con_contig08933	−52.6
	UCCCACAGCUUUCUUGAACUC	21		
miR418	AAUGAUGAUGAUGAUGAUGACG	22	bud_contig04831	−23.5
miR535	UGACAACGAGAAAGAGCACCC	21	bud_contig03834	−55.4
	UGACAACGAGAAAGAGCACGC	21		
	UGACAACGAGAAAGAGCACUC	21		
	UGACUACGAGAGAGAGCACGC	21		
	GACAACGAGAGAGAGCACGCUA	22		
	UGACAACGAGAGAGAGCACGCUA	23		
miR854	GAUGAGGAGGAGGAGGAGGAGGAG	24	con_contig03976	−167.3
miR900	AUGUGUUAUUGUAUCCUGGGAA	22	chi_contig14717	−21.5
	UCCCAGGAUACAAUAACACAUGA	23	chi_contig14717, bud_contig15809	−21.5, −86.9
miR1023	AGAGGAAGUGAGAGAGAGUGAU	22	con_contig20221	−80
miR1063	CAUCAUUGGAGUACUGAUGCAU	22	con_contig15919, chi_contig02825	−56.5, −41.5

*Contig IDs are used in the transcriptomic database. These IDs will be well-linked for searching despite further definitions being made.

### Precursor prediction of miRNA with transcriptomic database and secondary stem-loop structural simulation

**Figure 5:**
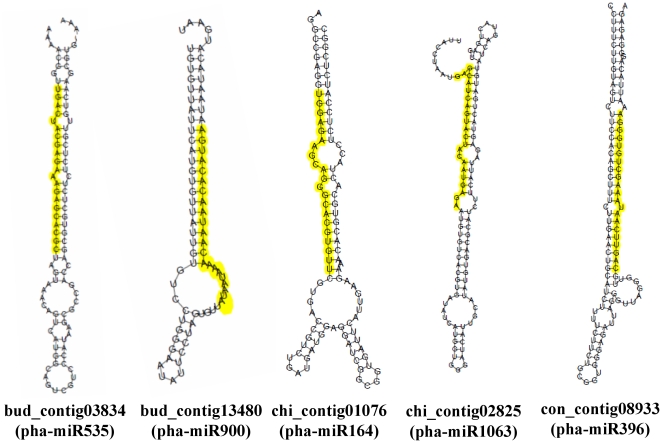
Secondary structure simulation of predicted miRNA precursors. pha: abbreviation for *Phalaenopsis aphrodite* subsp. *formosana*.

Pre-miRNAs have an important feature that distinguishes them from other small RNAs: they form a secondary hairpin structure. This characteristic offers a powerful approach for predicting the existence of new miRNA orthologs or homologs in other species [19]. To predict miRNA precursors, putative miRNA sequences were searched against a transcriptomic contig database (see Methods). Twenty-five of 11,129 identified miRNA sequences matched 33 transcript contigs that are thought to be primary miRNAs (pri-miRNAs). The secondary structure of these contigs were analyzed using the RNAfold web server [20]. Only 13 contigs belonging to 10 miRNA families had a putative secondary structure (Table 3). MFE (minimal folding free energy) was used to evaluate the stability of simulated RNA secondary structures. Generally, the lower the MFE, the more stable the formed RNA secondary structure. Stem-loop hairpin structures including mature miRNA sequences on both strands were used to calculate MFE. The average length of the hairpin structures was approximately 100 nucleotides. Because the input sequences differed in length, the range obtained for the MFE of these structures was −21.5 to −302.42 kcal/mol. Owing to their well-formed secondary structures, two pri-miRNA sequences belonging to miR396 and miR535 were considered potential candidates for further study (Figure 5).

**Table 3:** Abundant miRNAs present in *Phalaenopsis aphrodite* subsp. *formosana*.

miRNA family	Sequence	Length	Reads/clean reads (million)*				Relative change**
			NL	CL	S	FB	
miR156/157	**C**UGACAGAAGAUAGAGAGCAC	21	1131	3026	100	720	2.68
	UGACAGAAGAGAG**U**GAGCAC	20	490	1242	272	557	2.54
miR159	UUUGGAUUGAAGGGAGCU**CUA**	21	62	106	63	201	1.71
	UUUGCAUAACUCAGGAGCUGG	21	63	148	293	373	2.37
miR162	UCGAUAAACCUCUGCAUCCGG	21	47	70	32	93	1.48
miR164	UGGAGAAGCAGGGCACGUG**UU**	21	94	132	1	8	1.41
	UGGAGAAGCAGGGCACGUG**CA**	21	496	589	132	692	1.19
	UGGAGAAGCAGGGCAC**A**UGUU	21	36	51	2	13	1.41
miR166	**U**UCGGACCAGGCUUCAUUCCC	21	41	44	9	47	1.07
	UCGGACCAGGCUUCCUUCCCC	21	239	91	81	113	0.38
	UCGGACCAGGCUUCAUUC**CCC**	21	4409	4305	1428	4076	0.98
	GGAAUGUUGUCUGGCUCGAGG	21	20	62	72	77	3.07
	CGGACCAGGCUUCAUUCC**CC**	20	83	106	32	113	1.28
miR167	UGAAGCUGCCCGCAUGAUCUGA	22	19	19	0	1	0.99
	UGAAGCUGCCAGCAUGAUCUGU**UC**	24	1145	1358	41	82	1.19
	UGAAGCUGCCAGCAUGAUCUG**AA**	23	4314	4749	64	401	1.10
	**U**GAAGCUGCCAGCAUGAUCU**A**	22	310	365	447	406	1.18
miR168	UCGCUUGGUGCAGGUCGGGAU	21	461	977	232	352	2.12
	UCGCUUGGUGCAGGUCGGGAC	21	2061	4600	2730	2970	2.23
	**U**CGCUUGGUGCAGGUCGGGAA**U**	22	2436	3032	1573	1498	1.24
miR172	**C**GAAUCUUGAUGAUGCUGCAU	21	1401	652	260	169	0.47
	AGAAUCUUGAUGAUGCUGCAU	21	31	46	1840	1078	1.51
	AGAAUCAUGAUGAUGCUGCAA	21	277	123	59	332	0.44
miR390	AAGCUCAGGAGGGAUAGCGCC	21	41	38	24	26	0.93
miR396	**G**UUCAAUAAAGCUGUGGGAAA	21	35	57	4	14	1.63
	**G**UUCAAGACAGCUGUGGGAAA	21	57	109	46	83	1.91
miR528	UGGAAGGGGCAUGCAGAGGCG	21	14	38	2	32	2.71
	UGGAAGGGGCAUGCAGAGG**AGA**	22	6019	20583	1077	16462	3.42
	GGAAGGGGCAUGCAGAGGAG	20	177	684	32	528	3.87
miR535	**U**UGACAAAGAGAGAGAGCACG**C**	22	2019	3616	628	5578	1.79
	UGACAAAGAGAGAGAGCACGC	21	205	195	8	21	0.95
miR894	GUUUCACGUCGGGUUCAC**CA**	20	300	369	193	264	1.23

*Reads of each group is normalized with clean reads in million unit.

**Relative change = reads of low temperature-treated leaves (CL)/untreated group (NL). NL: leaves from untreated plants, CL: leaves from low ambient temperature treatment, S: stalks, FB: flower buds.

### Combination analyses reveal the major classes of miRNA families in response to low temperature in *Phalaenopsis aphrodite* subsp. *formosana*

**Figure 6:**
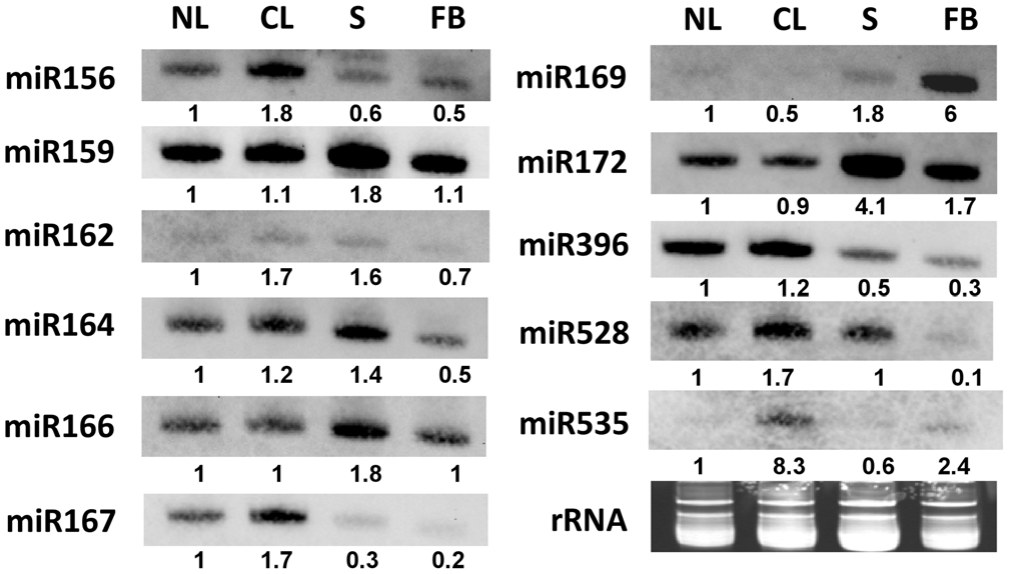
Analysis of miRNA expression levels in *Phalaenopsis aphrodite* subsp. *formosana* using northern hybridization. NL: leaves from untreated condition, CL: leaves from low ambient temperature treatment, S: stalks, FB: flower buds.

To elucidate the physiological roles of the abundant miRNAs and to validate these with the sequencing results, small RNA northern hybridization was performed for 11 miRNA families with samples from leaves, stalks and flower buds, or low temperature-treated leaves. The results indicated that miR156, miR162, miR167, miR528 and miR535 were induced in leaves under low ambient temperature treatment, while the expression levels of miR159, miR164, miR166, miR172 and miR396 remained unchanged, and that of miR169 was decreased (Figure 6). By comparing the results of direct sequencing and smRNA northern hybridization, four low temperature-responsive miRNA families were identified: miR156, miR162, miR528 and miR535.

Several miRNAs had different expression patterns in the leaf and inflorescent tissue. miR159, miR164 and miR166 accumulated in stalks, while miR172 accumulated in stalks and flower buds. miR156, miR167 and miR396 were expressed predominantly in leaves, whereas miR169 was expressed only in inflorescence tissue, particularly flower buds. Expression of miR167 and miR528 could not be detected in flower buds but were detected in other tissues (Figure 6). The results indicate the tissue specificity of expression and low ambient temperature-dependent response of miRNAs in *Phalaenopsis aphrodite* subsp. *formosana*. The number of sequence reads is thought to represent a quantitative measure of the expression of certain genes, as a result of the large amount of data generated from deep sequencing. In fact, the dynamic expression patterns obtained by small RNA blot analysis reflected the digital northern results from deep sequencing (Table 3, Figure 6).

### Identification and characterization of miRNAs potential targets in *Phalaenopsis aphrodite* subsp. *formosana*

To investigate the regulatory function of miRNA in *Phalaenopsis aphrodite* subsp. *formosana*, miRNA orthologs identified in *Phalaenopsis* orchid were compared against a deep sequencing-based transcriptomic database to identify target transcripts (see Methods). Through this bioinformatics analysis, hundreds of putative target transcripts were identified, and the number of target transcripts was lowered using abundant low temperature-responsive and tissue-specific miRNA sequences. BLASTX was performed with the selected target transcripts to identify the best homolog in *Arabidopsis* as molecular functions in this species are better understood. Several identified target transcripts are well-known miRNA target genes in *Arabidopsis* including the squamosa promoter-binding-like (*SPL*) genes of miR156, the MYB domain containing gene of miR159, the NAC-domain containing gene of miR164, PHABULOSA (*PHB*) of miR166, auxin responsive factors (*ARFs*) of miR167, ARGONAUTE 1 (*AGO1*) of miR168, scarecrow-like transcription factor of miR171, growth-regulating factors (*GRF*) of miR396 and laccase of miR397 (Table S2). The listed target transcripts from low temperature-responsive or tissue-specific miRNAs were further categorized according to molecular function and biological processes using gene ontology (GO) analysis. This analysis revealed that the majority of target transcripts were highly correlated with plant development and metabolic processes. Several of the well-annotated target transcripts such as *MYB65*, *NAC1*, *PHB* and *ARFs* had putative functions involved in floral organ formation (Table S2). The miRNAs of these genes accumulated in stalk but not in flower bud, with the exception of miR167, which may contribute specifically to floral transition or the differentiation of meristem (Figure 6).

**Figure 7:**
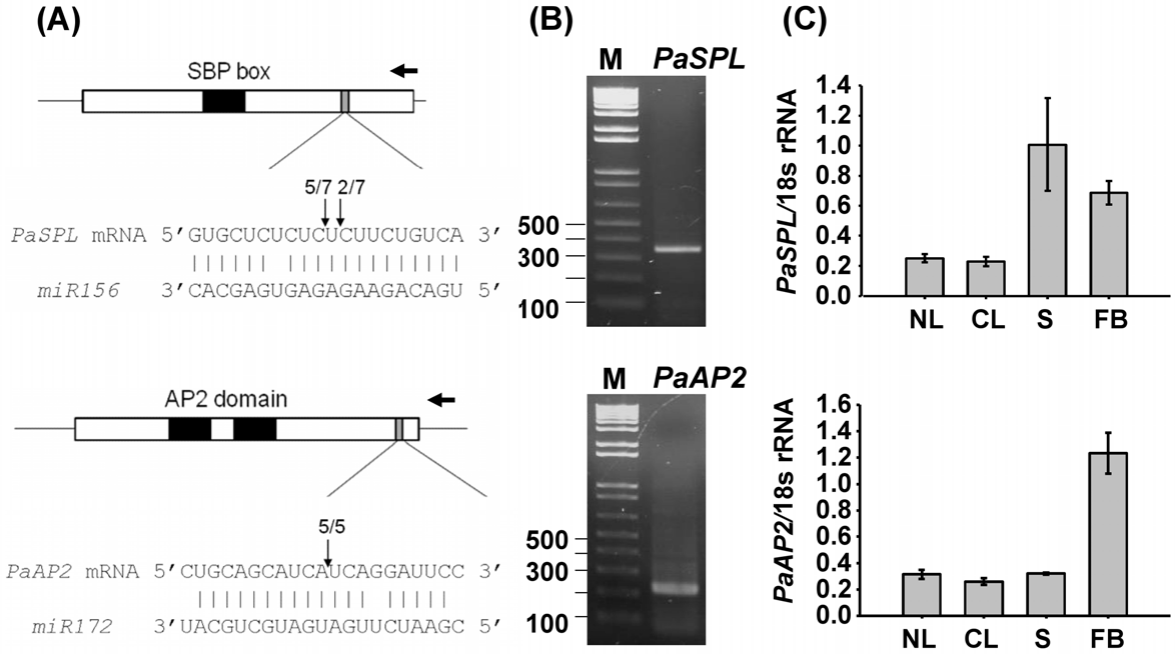
Identification of cleavage sites and expression patterns of *PaSPL* and *PaAP2* in *Phalaenopsis aphrodite* subsp. *formosana*. The arrows indicate the 5′ cleavage sites (A) that were sequenced from 5′-end cleavage products (B). The expressions of *PaSPL* and *PaAP2* were analyzed using Q-PCR. The 18S rRNA expression level was used as a quantitative control. NL: leaves from untreated condition, CL: leaves from low ambient temperature treatment, S: stalks, FB: flower buds.

For target genes of low temperature-responsive miRNAs, floral homeotic protein HUA1 is one newly predicted target gene of miR528. miR528 is almost absent in flower buds, suggesting the regulatory role concerns floral organ identity. In addition, miR156 and miR535 matched the same target transcripts and had similar expression patterns (Table S2, Figure 6). Using alignment, few gaps and no more than one mismatch were identified in the nucleotide sequences between miR156 and miR535, suggesting they may be somewhat functionally redundant in *Phalaenopsis*. Previous reports have determined that miR156 and miR172 play key roles in the transition from the vegetative to the reproductive phase, and they were determined to be low temperature-responsive in previous studies and in the present study. In order to validate that the suitability of the entire approach for investigating miRNA-mediated regulatory pathways, miR156- and miR172-mediated cleavage was confirmed by modified 5′-end RLM-RACE (RNA ligase mediated-rapid amplification of cDNA end) (Figure 7A and B) and the 5′-end cleavage products were successfully amplified. Furthermore, the expression patterns of *PaSPL* and *PaAP2* were analyzed. The expression of *PaSPL* was complementary to the expression of miR156, whereas *PaAP2* demonstrated inconsistent expression patterns (Figure 7C). A possible explanation of the fact that expression of *PaAP2* was not complementary to the expression of miR172 may be that miR172 contributes to its function via translational repression rather than by RNA cleavage [21].

## Discussion

This study is the first to identify small regulatory RNAs comprehensively in *Phalaenopsis aphrodite* subsp. *formosana*. Furthermore, it is the first to report that low ambient temperature-responsive miRNAs belong to four miRNA families and to identify their putative target transcripts. In addition, it showed that numerous sequences of miRNAs are conserved between *Phalaenopsis aphrodite* subsp. *formosana* and other plant species. Sixty-four, 58, 57 and 71 sequences were identified in untreated leaves, low temperature-treated leaves, stalks and flower buds, respectively, with perfect matches to known miRNAs present in other plant species (Table S3). Among them, the more abundant miRNA sequences were highly correlated with *Vitis vinifera*, in particular miR528, which has recently been characterized as a monocot-specific miRNA [22] originally discovered in *Oryza sativa* [23] and recently identified in another two *Poaceae* plants, *Zea mays* and *Sorghum bicolor* [24]. More than one thousand smRNA sequences were categorized as being in the miR528 family. The major sequence is 5′-UGGAAGGGGCAUGCAGAGGAG-3′, but there are more divergences in *Phalaenopsis* than in any other plant species (data not shown). miR535 is an unique miRNA first reported in moss (*Physcomitrella patens*) [25] and later identified in *Oryza sativa* and *Vitis vinifera* [26]. The miR528 and miR535 sequences identified were used in other species to search for target transcripts, but no similar target transcripts could be identified. miR528 was assumed to be a regulator of development because its expression increased during low ambient temperature treatment. Putative target homologs of miR528 have been reported to participate in nucleosome assembly, glycosylation, and ubiquitination and cell fate determination in other plant species [27], [28], [29]. Among these, HUA1 interacted with HEN4 and acted as a regulator of stamen and carpel identities by binding AGAMOUS pre-mRNA during RNA processing [27]. Another target transcript, histone H2A, was previously reported to participate in flower development via regulation of the expression of FLOWERING LOCUS C (FLC) in *Arabidopsis* [28].

Several studies have focused on the regulatory role of stress-responsive miRNAs. miR168, miR171 and miR396 were shown to be responsive to all stresses in *Arabidopsis* [8]. Many platforms have been employed for identifying low temperature-responsive miRNAs. However, for several miRNAs, the results of expression patterns were inconsistent among plant species (Table S4). Treatment at 16°C resulted in an increase in the expression of ath-miR156, ath-miR161.a2, ath-miR166 and ath-miR169, but decreased expression of ath-miR163, ath-miR172, ath-miR396∼399 and ath-miR830 [13]. Comparable results were generated in this study (Table S4). Lower temperature treatment (4°C) led to different miRNA expression patterns; in particular, the patterns of ath-miR156 and ath-miR172 were reversed, suggesting distinct regulatory roles for the two miRNAs in the response to ambient temperature and vernalization (near-freezing temperature) [8], [13]. Previous studies have demonstrated that miR156 is highly expressed during the juvenile phase and that expression gradually decreases in later developmental stages. *SPL* genes are negatively regulated by miR156 and function as positive regulators of *MIR172* genes. Accumulation of miR172 contributes to complete flowering through negative regulation of certain AP-2-like transcriptional factors such as *SMZ*, *SNZ*, *TOE1* and *TOE2* [30], but was not identified in the transcriptomic database in this study. Homologs of *SPL* genes were identified and reversed expression patterns were observed by Q-PCR analysis (Figure 7C). 3′- and 5′-RACE (rapid amplification of cDNA end) was performed and one AP-2 like transcript with a predicted miR172 target site was successfully obtained; however, the expression pattern was not completely reversed as would have been expected (Figure 7C). Previous studies have demonstrated that miR172 contributes its function via translational repression rather than by RNA cleavage [21], [31]. However, evidence from recent studies and herein suggests that miR172 also functions to degrade its target mRNAs [32], [33]. These results indicate that miR156- and miR172-mediated regulatory pathways in *Arabidopsis* and *Phalaenopsis* are similar, but more research is required to identify the downstream miR172-targeted genes that function in a similar manner to the known genes.

In this study, other small RNAs that may potentially function in regulating flowering time under low temperature treatment were not excluded (Figure 4B), and approximately 80% of abundant smRNAs consisted of currently unknown sequences. These sequences were grouped according to an increased or decreased ratio compared with low temperature-treated leaves and untreated leaves. The results revealed 74 smRNA sequences that could be classified into 28 groups. These smRNA sequences were searched against the transcriptomic database to identify novel miRNA precursors but none were identified. To understand the functional role of these undefined smRNAs, their putative target transcripts were investigated. Several smRNAs were completely complementary to their target transcripts including digalactosyldiacylglycerol synthase and alliin lysase, which may be siRNAs. However, no target transcripts that could be involved in flowering were identified. Most were involved in biosynthetic and metabolic processes (Table S5).

Individual miRNAs of one miRNA family had a distinct expression pattern (Table 2), suggesting that each individual miRNA may target specific transcripts to achieve unique functional inhibition. However, verifying the effects of the changes in sequences of miRNAs is technically challenging. smRNA hybridization may result in annealing of the probe to sequences containing 1 to 4 mismatches [34], [35]. Therefore, the results of smRNA hybridization led to combined expression patterns for each miRNA family. To increase sensitivity and specificity, it is suggested that future research should deploy smRNA sequence-based elongated primers designed for real-time quantitative PCR, particularly in the case of low-abundant smRNAs [35].

Few miRNA precursors could be identified in our transcriptomic database, possibly due to the loss of short RNA fragments isolated from total RNA using LiCl precipitation. Using LiCl precipitation would have avoided contamination with polysaccharides, polyphenol and other insoluble materials [36]. Furthermore, genomic or transcriptomic resources from dicer-mutated plants would be more efficient for identifying miRNA primary transcripts. Alternatively, the strategy of designing amiRNAs (artificial miRNA) rather than using native precursors for advanced studies concerning functional miRNAs is feasible [37], [38].

There are few EST sequences and one GSS (genome sequence survey) of *Phalaenopsis* orchids available in the public resources [39]. Among them, the information associated with miRNA is limited after analysis. Sequences of *Phalaenopsis* for bioinformatics analyses are more limited than for other model plants. High-throughput sequencing provides a more powerful way to establish a transcriptomic database than generating EST or genomic libraries. This study provides useful information for elucidating the regulatory mechanism underlying thermosensing and flowering time regulation.

## Materials and Methods

### Plants growth conditions

Seedlings of *Phalaenopsis aphrodite* subsp. *formosana* (M1624) were purchased from Chain Port Orchid Co., Ltd (Chengde Village, Taiwan) and grown in 2.5 in. diameter plastic pots filled with sphagnum moss in an environment-controlled greenhouse, at a constant temperature of 28°C for 7–8 months. Plants comprising the untreated group were subsequently grown at 32°C/27°C (light/dark) to inhibit phase transition. Low ambient temperature treatment was carried out at 24°C/18°C (light/dark) for two months to complete phase transition. Leaves with or without low temperature treatment were collected at 0.5 month intervals from zero to two months. Stalks and flower buds were only present in the low temperature treatment group. Stalks were collected at 1.5 and 2 months, while flower buds were collected at 3.5 months. The materials were harvested more than ten independent lines in each time point per batch and stored them separately for all the experiments in this study. We randomly picked out the individual plant materials and extracted the RNAs freshly before performing the experiments. We performed the biological replications for all of the experiments in this study.

### Small RNA library preparation and sequencing

Tissues frozen in liquid nitrogen were ground to fine powder using a mortar and pestle. Total RNA extraction was freshly prepared using Trizol (Invitrogen, Carlsbad, CA, USA) following the manufacturer's instructions. Small RNAs were size fractionized on a 15% denaturing polyacrylamide gel. Subsequently, gel bands corresponding to fragments in the size range of 20–25 nt were excised and eluted into 0.3 M NaCl. The eluted smRNAs were sequentially ligated to 5′- and 3′-adaptors and converted to DNA using RT-PCR. Products of RT-PCR were directly sequenced using an Illumina 1G Genome Analyzer according to the manufacturer's protocols (Beijing Genomics Institute, China) [40].

### Production of small RNA sequences

Raw data were processed by removing the adaptor/acceptor sequences, contaminants and low quality reads. To identify degradation tags of rRNA, tRNA, snRNA and snoRNA, sequences were aligned using Rfam 10.0 (http://rfam.sanger.ac.uk/) [16] and the Genbank database (http://www.ncbi.nlm.nih.gov/) [17]. The final dataset containing reads of orchid smRNA sequences were used for this study.

### Identification of conserved miRNA orthologs

Known miRNAs were downloaded from the miRBase15.0 database (http://www.mirbase.org/ftp.shtml) [41]. Alignment of fully complementary sequences, those with one mismatch or two mismatches, was performed using the BLAST algorithm to identify conserved miRNA orthologs. The number of aligned sequences was restricted by limiting the mismatch plus gap to fewer than 5 residues. The analyzed results were deposited in Gene Expression Omnibus (GEO) on NCBI website. The accession number is GSE27585. A comparison of *Phalaenopsis* miRNA sequences and miRNAs of other plant species is presented in the Supplemental Data.

### Establishment of a *Phalaenopsis* transcriptomic database

Freshly extracted total RNA was subjected to LiCl precipitation to reduce the effects from polysaccharides, polyphenols and other contaminants. Messenger RNAs were further isolated by binding to a polyA column. Following a subsequent ligation with adapters, samples were sequenced on a 454 pyrosequencing system. The results were incorporated into a sequence database that allowed stand-alone BLAST searches to be conducted.

### Precursor prediction and secondary structure analysis

Based on the feature of the stem-loop structure, a command line BLAST was performed to align each unique miRNA sequence with the sequences of the transcriptomic database, aligning forward and reverse strands separately by setting the parameter –S (1 for forward, 2 for reverse alignment). The conjunct contigs were selected from a combination of the aligned contigs of forward and reverse alignment results. Contigs that contained a coding sequence or more than 5 mismatches in the miRNA were excluded: miRNA* duplex. The selected contig sequences were filtered by secondary structure analysis. Sequences were submitted to the RNAfold web server (http://rna.tbi.univie.ac.at/cgi-bin/RNAfold.cgi) [20]. Contigs with correct stem-loop structure able to form a miRNA: miRNA* duplex were selected as putative miRNA precursors. The minimum free energy (MFE) was calculated using the stem-loop sequence producing the mature miRNA.

### Prediction of miRNA targets in *Phalaenopsis aphrodite* subsp. *Formosana*

Most plant miRNAs bind perfectly or nearly perfectly to complementary sites on their target genes. Several bioinformatic programs have been designed on the basis of this characteristic. To identify potential miRNA target genes in *Phalaenopsis aphrodite* subsp. *formosana*, the custom sequencing data were submitted to the psRNATarget web server (http://bioinfo3.noble.org/psRNATarget/) [42]. psRNATarget uses an iterative parallel Smith-Waterman-based algorithm. Default settings were used as analytic parameters, i.e. 3 as the threshold of maximum expectation, 2 as the number of the multiplicity of target sites, and 9 to 11 nt as the range of the central mismatched region. Outputs were generated into a MySQL database for web-based searching (provided as web source). smRNA biosynthesis pathway components were identified using the known protein sequences from *Arabidopsis* to perform tBlastn against the *Phalaenopsis* transcriptomic database (Table S1).

### Expression analysis of miRNA families

For detecting the expression of miRNA, 30 µg of total RNA was separated by 15% PAGE and transferred to a Hybond Nx membrane (GE Healthcare). The RNA was cross-linked to the membrane using carbodiimide [34]. End-labeled antisense DNA probes were prepared by adding biotin-dUTP to the 3′ ends of specific probes with TdT transferase (Genemark). A chemiluminescent nucleic acid detection module (Pierce) was used for visualization.

### Mapping of target gene cleavage sites and expression analysis of target genes expression by quantitative polymerase chain reaction (Q-PCR)

To map the cleavage sites in *PaSPL* and *PaAP2* mRNA, total RNAs were extracted from leaves, flower buds and flowers. The 5′-end of the cleavage product was determined by a modified 5′-RACE [43] using the RLM-RACE GeneRacer™ kit (Invitrogen, Carlsbad, CA, USA). The amplification conditions accorded with the manufacturer's instructions. The 5′-end of cDNA specific to the cleaved *PaSPL* and *PaAP2* mRNA was amplified with the GeneRacer™ 5′ Primer, gene specific primers PaSPL 5′ RACE (5′-TCAGGCCTTGAACCCCAGAAAGTGCCCA-3′), PaAP2 5′ I (5′-CACCCTCCCCACCTTAGAACAGAATGTTTGC-3′), and PaAP2 5′ II (5′-CTGAGAGGATAGAGGCGCTGTCATTTCTCT-3′).

Quantitative RT-PCR was performed using the SYBR green method and the ABI 7300HT real-time PCR system (Applied Biosystems). PaSPL-specific primers, 5′-GCACCACTGCTGAGGTCCTTAATCA-3′ (forward) and 5′-GGCGATGATCATATGGCTCTGTCC-3′ (reverse), PaAP2-specific primers, 5′-CATCTGTCATTGGGGATCGACGG-3′ (forward) and 5′-CCCCTTCTCTTTTGCAGCCAATTG-3′ (reverse), and 18s rRNA-specific primers 5′-CAGGCCCGGGTAATCTTGGG-3′ (forward) and 5′-CTTCACCGGACCATTCAATCGG-3′ (reverse). All primers of miRNA target genes were designed on the basis of the sequence of contigs. The 18S rRNA expression level was used as a quantitative control.

## Supporting Information

Table S1 List of smRNA biosynthesis pathway components identified in *Phalaenopsis* orchid.
(DOC)

Table S2 Summary table of target genes with contigs and functional categorization.
(XLS)

Table S3 Comparison of conserved miRNA families in plant species.
(XLS)

Table S4 Low temperature-responsive miRNA families in various plant species.
(DOC)

Table S5 Characteristics of undefined low ambient temperature-responsive small RNAs.
(XLS)
