# Supplementary material for: Sequencing-Based Approaches Reveal Low Ambient Temperature-Responsive and Tissue-Specific MicroRNAs in Phalaenopsis Orchid
Source: PLoS One. 2011 May 6;6(5):e18937. doi: 10.1371/journal.pone.0018937 (PMC3089612; doi:10.1371/journal.pone.0018937)
Supplement: Table S1 — List of smRNA biosynthesis pathway components identified in Phalaenopsis orchid. (DOC) [file pone.0018937.s001.doc]

Supplemental Table 1. Contig homologs of smRNA biosynthesis pathway components in *Phalaenopsis* orchid

| Gene Name | AT No. | Function | Contig homolog | sum of reads | CDS |
| --- | --- | --- | --- | --- | --- |
| AGO1 | AT1G48410.2 | RNA Slicer, selectively recruits microRNAs and siRNAs | con_contig11836, chi_contig14312, chi_contig02741, sta_contig04286, sta_contig11534, bud_contig15514 | 376 | partial |
| AGO7 | AT1G69440.1 | RNA slicer, Involved in the regulation of developmental timing. Required for the accumulation of TAS3 ta-siRNAs | ND | ND | ND |
| AIN1 | AT1G54490.1 | 5'-3' exonuclease/ nucleic acid binding | con_contig03312, chi_contig01811, sta_contig03326, bud_contig00715 | 110 | partial |
| CHR38 | AT3G42670.1 | Nuclear RNA silencing and the spread of a silencing signal between cells | con_contig01648, chi_contig10115, sta_contig09662, bud_contig00846 | 49 | partial |
| CMT3 | AT1G69770.1 | Histone H3-K9 methylation, DNA methylation on cytosine within a CNG sequence, negative regulation of gene expression, epigenetic | con_contig05000, sta_contig11224, bud_contig00055 | 33 | partial |
| DCL1 | AT1G01040.1 | RNase III | con_contig10240, chi_contig10659, sta_contig04911, bud_contig06301 | 24 | partial |
| DCL2 | AT3G03300.1 | RNase III | sta_contig10907, bud_contig03563 | 12 | partial |
| DCL3 | AT3G43920.3 | RNase III | sta_contig04066, bud_contig07932 | 10 | partial |
| DCL4 | AT5G20320.1 | RNase III | sta_contig08471, bud_contig03323 | 26 | partial |
| DML1 | AT2G36490.1 | A repressor of transcriptional gene silencing. Functions by demethylating the target promoter DNA. | chi_contig04998, sta_contig01877, bud_contig14383 | 24 | partial |
| DRB4 | AT3G62800.1 | dsRNA-binding protein | con_contig21888, chi_contig07308, sta_contig09515, bud_contig03843 | 64 | partial |
| DRD1 | AT2G16390.1 | DNA methylation | con_contig02196, chi_contig00746, sta_contig07733, bud_contig09433 | 126 | partial |
| DRM2 | AT5G14620.1 | Histone H3-K9 methylation, DNA methylation | con_contig14324, sta_contig06815, bud_contig07660 | 39 | partial |

Supplemental Table 1. *continued*

| Gene Name | AT No. | Function | Contig homolog | sum of reads | CDS |
| --- | --- | --- | --- | --- | --- |
| HDA6 | AT4G38130.2 | Gene silencing, histone deacetylation, PTGS | con_contig10624, con_contig08857, chi_contig08892, sta_contig00183, bud_contig05288 | 62 | partial |
| HEN1 | AT4G20910.1 | RNA methylase | con_contig13183, sta_contig05055, bud_contig13155 | 22 | partial |
| HST | AT3G05040.1 | Exportin-5 otholog | chi_contig09454, sta_contig04619, bud_contig10635 | 29 | partial |
| HYL1 | AT1G09700.1 | dsRNA-binding protein | con_contig02293, chi_contig03939, sta_contig01277, bud_contig04215 | 75 | partial |
| MET1 | AT5G49160.1 | maintenance of DNA methylation, DNA methylation on cytosine within a CG sequence | sta_contig00402, bud_contig11025 | 41 | partial |
| NRPD1a | AT1G63020.1 | DNA-dependent RNA polymerase | con_contig23243, chi_contig00418 | 1486 | partial |
| NRPD1b | AT2G40030.1 | DNA methylation, PTGS | con_contig10002, sta_contig01350, bud_contig04833 | 630 | partial |
| RDR2 | AT4G11130.1 | RNA-dependent RNA polymerase | con_contig04705, chi_contig02030, sta_contig05112, bud_contig02751 | 219 | partial |
| RDR6 | AT3G49500.1 | RNA-dependent RNA polymerase | con_contig03783, chi_contig03816 | 41 | partial |
| SDE3 | AT1G05460.1 | RNA helicase | con_contig14050, chi_contig03176, sta_contig09171, bud_contig08684 | 85 | partial |
| SE | AT2G27100.1 | Response of leaf development to light | con_contig18420, chi_contig12680, sta_contig00235, bud_contig00074 | 64 | partial |
| SGS3 | AT5G23570.1 | RNA stabilizer | con_contig01414, chi_contig03473, sta_contig02658, bud_contig00928 | 177 | partial |
| SUVH4 | AT5G13960.1 | Histone H3-K9 methylation, maintenance of DNA methylation | ND | ND |  |
| SUVH5 | AT2G35160.1 | Chromotion silencing by smRNA, maintenance of DNA methylation, histion methylation | con_contig16621, chi_contig01504, sta_contig08596, bud_contig10946 | 134 | partial |
| SUVH6 | AT2G22740.2 | Histone methylation | sta_contig04414, bud_contig01198 | 33 | partial |
| WRNEXO | AT4G13870.2 | RNaseD exonuclease | con_contig09016, chi_contig14936, sta_contig10773, bud_contig06984 | 38 | partial |

ND: not detected.
