# Supplementary material for: Sequencing-Based Approaches Reveal Low Ambient Temperature-Responsive and Tissue-Specific MicroRNAs in Phalaenopsis Orchid
Source: PLoS One. 2011 May 6;6(5):e18937. doi: 10.1371/journal.pone.0018937 (PMC3089612; doi:10.1371/journal.pone.0018937)
Supplement: Table S4 — Low temperature-responsive miRNA families in various plant species. (DOC) [file pone.0018937.s004.doc]

**Supplemental Table 4.** Low temperature responsive miRNA families in different plant species

| miRNA family | *Phalaenopsis*  Sequencing  (this study) | *Phalaenopsis*  Northern  (this study) | *Arabidopsis thaliana*  (Lee et al. 2010) | *Oryza sativa* microarray  (Lv et al. 2010) | *Populus*  microarray  (Lu et al. 2008) | *Brachypodium*  sequencing  (Zhang et al. 2009) |
| --- | --- | --- | --- | --- | --- | --- |
| miR156/157 | 🡩 | 🡩 | 🡩 | 🡫 | 🡫 |  |
| miR159 | 🡩 | − |  |  |  |  |
| miR162 | 🡩 | 🡩 |  |  |  |  |
| miR164 | − | − |  |  |  |  |
| miR166 | − | − | 🡩 | 🡩 |  |  |
| miR167 |  | 🡩 |  | − |  |  |
| miR168 | 🡩 |  |  | 🡫 | 🡩 |  |
| miR169 | − | 🡫 | 🡩 | 🡫 | 🡩 | 🡩 |
| miR172 | 🡫 | − | 🡫 |  |  | 🡩 |
| miR396 | 🡩 | − | 🡫 |  | 🡩 |  |
| miR528 | 🡩 | 🡩 |  |  |  |  |
| miR535 | 🡩 | 🡩 |  | 🡩 |  |  |
| miR894 | 🡩 |  |  |  |  |  |

Lee H, Yoo SJ, Lee JH, Kim W, Yoo SK, Fitzgerald H, Carrington JC, Ahn JH. 2010. Genetic framework for flowering-time regulation by ambient temperature-responsive miRNAs in Arabidopsis. *Nucleic Acids Res* *38*:3081-3093.

Lu S, Sun YH, Chiang VL. 2008. Stress-responsive microRNAs in Populus. *Plant J* *55*:131-151.

Lv DK, Bai X, Li Y, Ding XD, Ge Y, Cai H, Ji W, Wu N, Zhu YM. 2010. Profiling of cold-stress-responsive miRNAs in rice by microarrays. *Gene* *459*:39-47.

Zhang J, Xu Y, Huan Q, Chong K. 2009. Deep sequencing of Brachypodium small RNAs at the global genome level identifies microRNAs involved in cold stress response. *BMC Genomics* *10*:449.
